# Blast‐Overpressure Induced Modulation of PARP‐SIRT‐NRF2 Axis in Stress Signaling of Astrocytes and Microglia

**DOI:** 10.1002/iid3.70106

**Published:** 2025-01-27

**Authors:** Vijaya Prakash Krishnan Muthaiah, Kathiravan Kaliyappan, Ramkumar Thiayagarajan, Supriya Mahajan, Krishnamoorthy Gunasekaran

**Affiliations:** ^1^ Department of Rehabilitation Sciences, School of Public Health and Health Professions University at Buffalo Buffalo New York USA; ^2^ Department of Geriatric Medicine, Kansas University Medical Center The University of Kansas Kansas City Kansas USA; ^3^ Department of Medicine, Division of Allergy, Immunology & Rheumatology SUNY University at Buffalo Buffalo New York USA; ^4^ Department of Medical Biochemistry, Institute of Health Dambi Dollo University Dambi Dolo Ethiopia

**Keywords:** ATP, blast‐overpressure, Glia, poly ADP‐ribose polymerase, sirtuins

## Abstract

**Background:**

The pathomechanism of blast traumatic brain injury (TBI) and blunt TBI is different. In blast injury, evidence indicates that a single blast exposure can often manifest long‐term neurological impairments. However, its pathomechanism is still elusive, and treatments have been symptomatic. Poly adenosine diphosphate (ADP) ribose polymerase‐1 (PARP1) is implicated in the parthanatos and secondary neuroinflammation. Animal studies indicate the over‐activation of PARP1 as a significant downstream event underlying the neurological sequelae of several traumatic and neurodegenerative disorders, irrespective of the mode of cell death. PARP over‐activation forms ADP polymers on several nuclear proteins, known as trans‐PARylation, by consuming nicotinamide adenine dinucleotide (NAD^+^) and ATP. As NAD^+^ is a substrate for sirtuins, ithas also been implicated in the oxidative stress underlying TBI pathology.

**Hypothesis:**

We recently established the implication of PARP1 following blast overpressure (BOP) and its differential response on astrocytes and microglial cells. We found that the inhibition of PARP is proven beneficial by attenuating oxidative stress. In this study, we hypothesized the involvement of the PARP1‐SIRT‐NRF2 axis following induced blast‐induced PARP over‐activation in glial cells for the manifestation of oxidative stress in BOP insults.

**Objective:**

The objective is to determine the downstream modulation of the PARP‐SIRT‐NRF2 axis and changes in ATP levels following blast exposure in astrocytes and microglia cell lines.

**Results:**

As a result of NAD^+^ being a common substrate for PARP1 and Sirtuins, we found the decreased expression of SIRT1, SIRT3, and NRF2, a central transcriptional regulator for the expression of antioxidant genes. We found that ATP levels were elevated post‐BOP from both glycolysis and oxidative phosphorylation (OXPHOS), an increase of ATP by glycolysis more significant than OXPHOS source, indicating the proinflammation post‐BOP.

**Conclusion:**

This result shows that blast‐induced PARP1 over‐activation impacts the deacetylation activity of sirtuins and consequently impacts the regulation of antioxidant levels in astrocytes and microglia.

## Graphical abstract

In blast injury, ATP levels were elevated from both glycolysis and oxidative phosphorylation (OXPHOS). An increase of ATP by glycolysis is more significant than the OXPHOS source indicating the proinflammation in post‐BOP. Overall, blast‐induced PARP over‐activation impacts the deacetylation activity of sirtuins and the regulation of antioxidant levels in astrocytes and microglia.

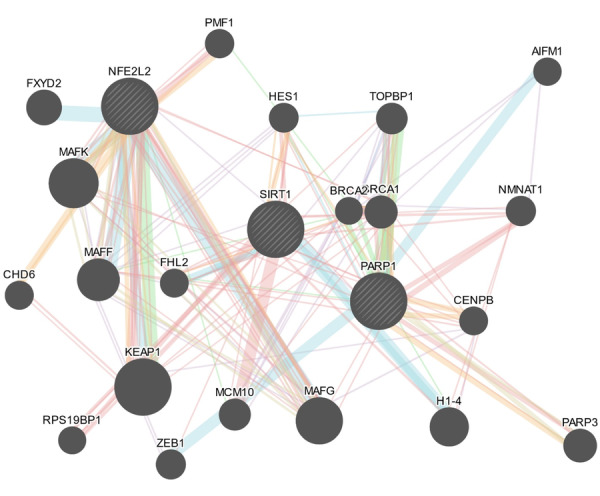

## 1 Introduction

The combat deployed veterans suffering blast concussive injury manifest neurological impairments even after years of mild insult [1]. These manifestations even lead to the consideration of mental health as a TBI marker of prognosis for blast injury [2, 3]. It is evidenced that a single blast could lead to the manifestation of early onset of neurodegenerative disorders. Hence, it is essential to investigate the cellular mechanism of blast TBI. Cell death mechanisms and molecular pathways following TBI have been studied [4, 5, 6] by several experimental invitro and invivo models of TBI. These in vivo and in vitro models were investigated using controlled cortical impact injury, which translates to the impact or inertial TBI. Given the nature of the insult, the TBI pathomechanism of impact injury (Blunt trauma) is different from nonimpact injury (Blast trauma). However, even the blast overpressure specifications of previously reported in vitro models of primary blast injury [7, 8, 9, 10, 11, 12, 13, 14] were of high frequencies in nature. In the present study, conforming to the ideal Friedlander equation of blast wave (with a duration of impulse wave < 2 ms followed by a negative pressure phase), we used a blast set‐up with a power spectral density of blast overpressure (BOP) energy lying within several kHz below 10 kHz [15, 16] to have a better mechanistic insight on cellular pathomechanism post‐blast TBI to translate to semi‐quasi real‐world Improvised Explosive Devices (IED) conditions.

Poly ADP Ribose Polymerase‐1 (PARP1), a nuclear enzyme, being a key mediator of cell death and its over‐activation, has been reported in both in vivo and in vitro models of several neurodegenerative disorders and traumatic insults. PARP‐induced PAR (Poly ADP Ribose) polymer formation results in NAD and Adenosine Triphosphate (ATP) depletion, mediating cell death through the release of Apoptosis‐Inducing Factor (AIF), often referred to as Parthanatos. However, evidence is confounding of regarding the mode of cell death as its over‐activation is implicated in both apoptosis [17] and necrosis [18] with overlapping signaling cascades. In addition, several proteases (such as caspase, cathepsin, and calpains) induced differential cleavage of PARP is reported following insults [19, 20]. Evidence indicates that PARP cleavage inhibits the necrotic mode and paves the way for cell death by apoptosis [21]. As NAD availability determines the level of Sirtuin (Silent information regulator) activation, it was reported that PARP1 activation reduces Sirtuin (SIRT1) activity and influences oxidative metabolism [22]. Sirtuins are predominantly NAD‐dependent lysine deacetylases (SIRT1, 2, 3 and 5) and ADP ribosylases (SIRT4, 6 and 7) [23] in addition to the function of various posttranslational modifications on a spectrum of targets, that are localized in various cellular compartments such as nucleus (SIRT1, 6 and 7), mitochondria (SIRT3, 4 and 5) and cytoplasm (SIRT2) [24, 25]. Sirtuins influence the levels of reactive oxygen species and antioxidant enzymes by regulating the mitochondrial electron transport complexes [26] through the Foxo3a transcription factor [27]. NRF2 (Nuclear Factor erythroid‐derived 2 like ‐related factor 2) is one of the significant targets of Sirtuins, NAD‐dependent class 3 histone deacetylases. NRF2 is typically labeled for proteasomal degradation by coupling with CUL3 and KEAP1 [28]. However, acetylation of NRF2 by sirtuins enhances the DNA binding capacity resulting in increased r mRNA expression of several antioxidant enzymes by binding to Antioxidant Response Element (ARE) regions of DNA. Zander et al. 2015 studied the outcome of single blasts and repeated blast and repeated blasts on dissociated neurons. As we demonstrated PARP overactivation post‐blast exposure, in the present study, we investigated the cellular pathomechanism of blast injury on the PARP‐SIRT‐NRF2 axis using in vitro model of low‐frequency blast injury using astrocytes (C6) and microglial cells (HTHU).

## 2 Materials and Methods

### 2.1 Cell Culture and Blast Injury

**Figure 1:**
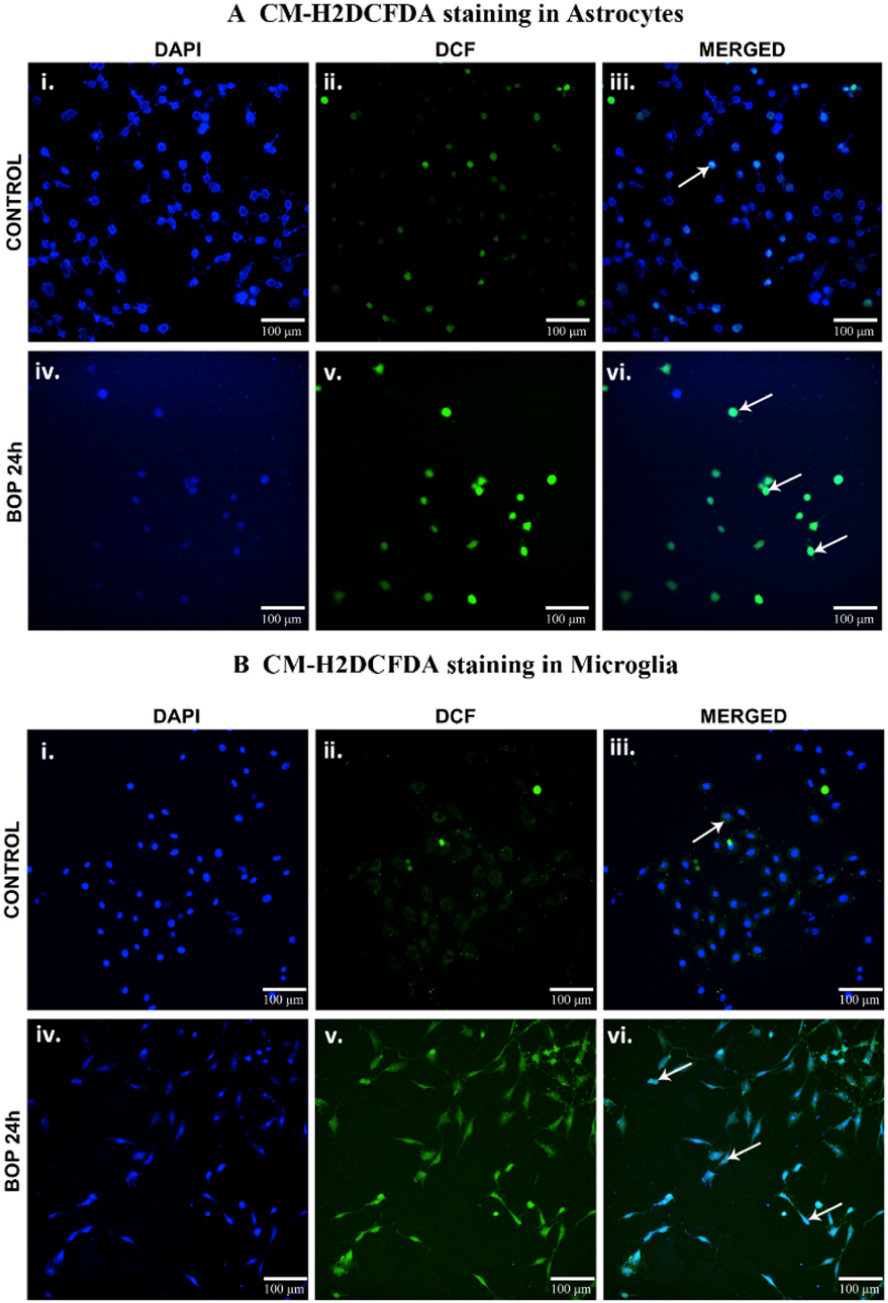
Qualitative assessment of CM‐H2DCFDA relative fluorescence in C6 and HTHU cells after BOP exposure. The images display the cells expressing the nuclear stain DAPI (i and iv), ROS marker CM‐H2DCFDA (ii and v) and merged images (iii and vi) in control and 24 h post‐BOP (A) C6 and (B) HTHU cells. The white arrows indicate the cells expressing the CM‐H2DCFDA with nuclear stain DAPI in C6 and HTHU cells.

The Rat C6 (ATCC CCL‐107) and HTHU cells (From Jonathan Karn, CWRU, Cleveland, OH) were cultured at DMEM (Corning, 10‐017‐CV)/F12 (Corning, 10‐080‐CV) 114 medium supplemented with 10% fetal bovine serum (Sigma, F2442) and 1% penicillin‐streptomycin (Corning, 30‐002 Cl). The acoustic shock tube generates blast over‐pressure (BC Precision Tools Inc) using compressed air at 23 psi is used to create an in‐vitro model of blast injury similar to our previous reports [15, 16]. The frequency spectrum of the impulse waveform is represented in Figure 1. Cells cultured at 12 well plates and 100 mm Petri dishes were exposed to single blast over‐pressure at ~172 dB SPL (decibel sound pressure level). Postinjury, the cells were maintained at a humidified air incubator (Fisher Scientific 610) at 37°C and 5% CO_2_ for 6, 12, and 24 h for various assays to investigate the effect of blast PARP‐Siirt‐NRF2 axis.

### 2.2 CM‐H2DCFDA Staining for ROS Detection

Reactive oxygen species generation in C6 and HTHU cells after BOP was visualized using the method described earlier [29]. To visualize the ROS in live cells, the cultured cells were stained using the CM‐H2DCFDA (LifeTechnologies) with 4′,6‐diamidino‐2‐phenylindole (DAPI). CM‐H2DCFDA is a chloromethyl derivative of 2′,7′‐Dichlorofluorescin Diace. Once it passively diffused into cells, its acetate groups are intracellular esterases. It's oxidation and the resulting fluorescent adduct indicates the ROS level. In brief, the C6 and HTHU cells (20,000 cells/plate) were seeded in a 15 mm glass‐bottom cell culture dish (NEST, Cat# 801002) exposed to BOP. Post 24 h, the cells were incubated with 13 µM CM‐H2DCFDA in a culture medium at 37°C for 10 min. After incubation, the cells were carefully washed thrice with warm PBS, then the cells were counterstained with DAPI (10 µg/mL) in PBS for 10 min and immediately imaged. The ROS in the cells were observed as a fluorescent emission of CM‐H2DCFDA (excitation/emission: 495/530 nm) with DAPI (excitation/emission: 358/461 nm).

### 2.3 Analysis of Mitochondrial Membrane Potential (JC10 Assay)

The Cell Meter JC‐10 Assay Kit (AAT Bioquest) assessed the mitochondrion membrane potential after BOP. The cells were seeded (1 × 104/well) in 96 well plates, cultured for 12 h, and exposed to the BOP. After 24 h post‐BOP, the treated cells were added with 50 μL/well of JC‐10 dye‐working solution (1 part of Component A + 100 parts of Component B) and incubated at 37°C in the dark. After 60 min, 50 μL of Component C was added to each well before reading the fluorescence intensity. The fluorescence intensities were measured on a microplate reader (BioTek, USA) using dual fluorescence (Ex/Em = 490/525 nm, Cutoff = 515 nm, and 540/590 nm, Cutoff = 570 nm) with bottom read mode. The ratio of green/red was calculated to denote the extent of mitochondrial depolarization.

### 2.4 Cellular and Mitochondrial ATP Production

Non‐mitochondrial cellular ATP production (glycoATP) through glycolytic pathway and mitochondrial ATP production (mitoATP) through oxidative phosphorylation (OXPHOS) was quantified using XFp Real‐Time ATP rate assay (Agilent Technologies, Santa Clara, USA). Proton efflux rate (PER) through the glycolytic pathway is used to measure cellular ATP, whereas the difference in oxygen consumption (Oxygen consumption rate, OCR) after the addition of mitochondrial inhibitors (oligomycin, inhibits Complex V and Rotenone/Antimycin mix inhibits Complex I/III) are used to measure mitochondrial ATP. The sum of glycoATP and mitoATP provides the total cellular ATP production. In brief, 5 × 105 cells were seeded into 100 mm Petri‐dish 12 h before the BOP. Both sham control and BOP‐exposedtoBOP‐exposed C6 and HTHU cells were subjected to real‐time ATP rate assay after different time points (6, 12, and 24 h) by transferring them to a calibrated seahorse XF tissue‐culture plate (5 × 104 cells/well). The basal extracellular acidification rate (ECAR) and OCR were analyzed in real‐time (Sea horse XFp analyzer) in the Seahorse assay medium supplemented with 10 mM glucose, 2mM l‐glutamine, and 1 mM pyruvate. After adding one µM oligomycin and a mix of 0.5 µM rotenone/antimycin‐A, mitochondrial OCR was measured. The sample triplicate was used in all experiments.

### 2.5 mRNA Expression of SIRT1, SIRT3, NRF2, and GCLC

The expression levels of SIRT1, SIRT3, NRF2, and GCLC genes following BOP were analyzed by quantitative polymerase chain reaction (qPCR). The RNA from the C6 and HTHU cells were extracted using TRIzol Reagent (Cat# 15596026; Thermo Fisher Scientific, USA) and assessed in NanoDrop One (Thermo Scientific, USA) for its purity and concentration. guide according to the manufacturer's guide, the High‐Capacity cDNA Reverse Transcription Kit (Cat# 4368814; Thermo Fisher Scientific, USA) was used to synthesize cDNA from the extracted RNA samples. The qPCR was performed in the CFX96 Touch Real‐Time PCR Detection System (Bio‐Rad, USA). The thermal cycler reaction conditions were set as follows: Polymerase Activation and DNA Denaturation for 30 s at 95°C; Denaturation for 15 s at 95°C; Annealing/Extension for 30 s at 60°C. The reaction mixture was prepared by adding the cDNA template, forward and reverse primers (Integrated DNA Technologies Inc. USA), and 2× SsoAdvanced Universal SYBR Green Supermix (Bio‐Rad, USA). The primer details are listed in Table 1. The GAPDH was used as the internal control for the assessment. After 40 reaction cycles, the relative gene expression was calculated using the standard curve of cycle thresholds (CT). In brief, the results were analyzed by the relative quantification method (2^−ΔΔCT^) using CFX Manager software (Bio‐Rad, USA). The specific products were confirmed by analyzing the melt curves. The experiment was carried out using sample triplicates.

**Table 1:** List of primers.

Name	Primer details	Primer sequence (C6)	Primer sequence (HTHU)
SIRT1	Forward primer	5′GAAACCCTCAATTTCTGTTCTGCT3′	5′GGGCTGCGGTTCCTACTG′3
	Reverse primer	3′AATGCGATGCTGACTTCCTTCT5′	5′TTATCTGGCTGCTGCGGAAA′3
SIRT3	Forward primer	5′GGCACTACAGGCCCAATGTC3′	5′GGTAGTTGAACGGGTCGAGG3′
	Reverse primer	3′TCTCTCAAGCCCGTCGATG5′	5′TAATAATCGTCCCTGCCGCC3′
NRF2	Forward primer	5′CCCAGCACATCCAGACAGAC3′	5′AGGTTGCCCACATTCCCAAA′3
	Reverse primer	3′TATCCAGGGCAAGCGACTC5′	5′AGGTTGCCCACATTCCCAAA′3
GCLC	Forward primer	5′TGCACATCTACCACGCAGTC3′	5′GTTGAGGCCAACATGCGAAA′3
	Reverse primer	3′ACCAACATGTACTCCACCTCG5′	3′TGTTAAGGTACTGGGAAATGAAGT′5
GAPDH	Forward primer	5′CCGCATCTTCTTGTGCAGTG3′	5′AAAGCCTGCCGGTGACTAAC′3
	Reverse primer	3′CGATACGGCCAAATCCGTTC5′	3′AGGAAAAGCATCACCCGGAG′5

### 2.6 Western Blot Analysis ‐ Protein Expression of SIRT1, SIRT3

The western blot analysis procedure assessed the protein levels of SIRT1 and SIRT3 in C6 and HTHU cells. In brief, the BOP‐exposedBOP‐exposed C6 and HTHU cells were washed twice with PBS and added with RIPA lysis buffer (Cat# sc‐24948; Santa Cruz Biotechnology, USA) at desired time points (6, 12 and 24 h), and the cell lysate was collected. The total protein levels in the samples were measured using the Pierce BCA Protein Assay kit (Cat# 23227; Thermo Fisher Scientific, USA). The proteins were separated by SDS‐PAGE by adding 30 µg of proteins in each well of 10% precast Tris‐glycine gels with the protein Standards (Cat# 1610374; BioRad, USA). Then, the separated proteins were transferred into the PVDF Transfer Membrane (Cat# 88518; Thermo Fisher Scientific, USA) using the Wet transfer method. The blots were incubated in 5% BSA for 1 h at room temperature to reduce the nonspecific binding. Following the blocking procedure, the blots were incubated in Anti‐SIRT1 Rabbit mAB (1:2000) (Cat# 9542; Cell Signaling Technology, USA), Anti‐ SIRT3 Rabbit mAB (1:2000) (Cat# 83732; Cell Signaling Technology, USA) in 4°C for overnight. Then, the blots were washed and incubated in pre‐diluted HRP conjugated Anti‐Rabbit secondary antibodies (1:5000) (Cat# 7074; Cell Signaling Technology, USA) for 1 h at room temperature. The blots were imaged in ChemiDoc MP Imaging System using SuperSignal West Femto Maximum Sensitivity Substrate (Cat# 34095; Thermo Fisher Scientific, USA). The protein band intensities were measured using Image Lab Software (BioRad, USA) and normalized against the internal control protein, βActin (Cat# sc‐47778; Santa Cruz Biotechnology, USA).

## 3 Results

### 3.1 BOP Increases Reactive Oxygen Species

The reactive oxygen species levels in the C6 and HTHU cells were qualitatively analyzed using CM‐H2DCFDA staining. It is a useful fluorescent indicator that passively diffuses into the cells and cleaved by the intracellular esterases and oxidation. The fluorescent signal identified increased ROS levels in the cells. The fluorescent signal (Em: 517–527 nm) identified increased cell ROS levels upon excitation with appropriate wavelength light (Ex: 492–495 nm). The results showed increased fluorescence (arrows) in C6 and HTHU cells at post‐BOP 24 h (Figure 1). The increased fluorescence indicates increased cell oxidation following BOP exposure [30].

### 3.2 Mitochondrial Membrane Potential (JC‐10 Assay)

**Figure 2:**
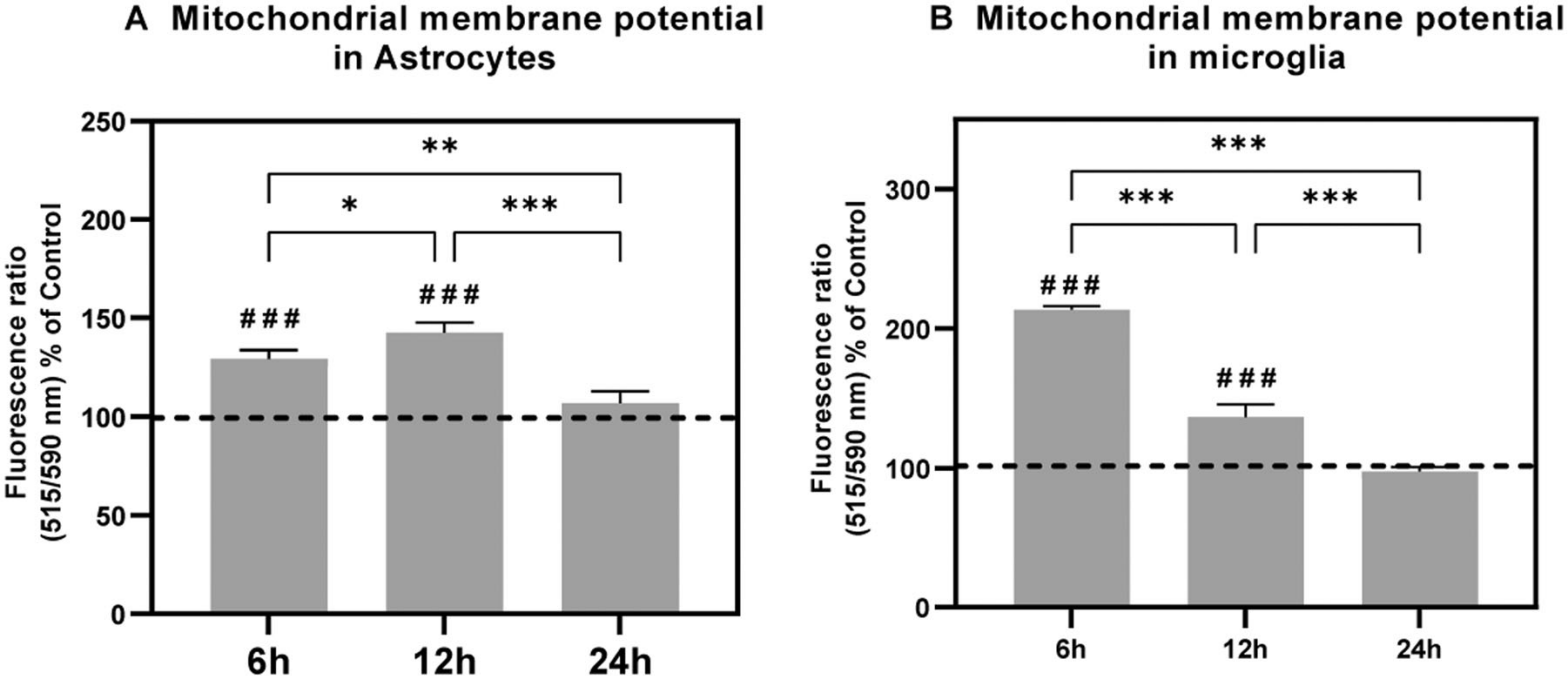
Mitochondrial membrane potential after blast overpressure. The histograms show the mitochondrial membrane potential (JC10) in (A) C6 cells and (B) HTHU cells in various timepoints (6, 12 and 24 h) following BOP. Statistical difference across different timepoints in BOP group (*p < 0.05 vs. 6 h; **p < 0.005 vs. 12 h; ***p < 0.001 vs. 24 h) and from different groups were indicated (###*p* < 0.001 vs Sham). Values are represented as Mean ± SEM (*n* = 3).

The mitochondrial membrane potential in BOP‐exposed C6 and HTHU cells was measured using spectrophotometric analysis by measuring the JC‐10 aggregates because of membrane polarization. The results indicate a significant increase in the membrane polarization following blast exposure (Figure 2). The JC‐10 aggregates were significantly increased (*F* (1, 12) = 79.56; *p* < 0.001) in BOP 6 h and BOP 12 h exposed C6 cells when compared with the sham group. Among BOP groups, the BOP 6 h (*F* (2, 8) = 22.06; *p* < 0.01) and BOP 12 h (*F* (2, 8) = 22.06; *p* < 0.001) group C6 cells have significantly higher membrane polarization when compared to post‐BOP 24 h group. Similarly, the HTHU cells also showed a significant increase (*F* (1, 12) = 217.2; *p* < 0.001) in membrane polarization at BOP 6 h and BOP 12 h groups when compared to the sham‐exposed cells. The HTHU cells showed a time‐dependent decrease in membrane polarization in BOP‐exposed cells. The six h post‐BOP exposed HTHU cells showed significantly increased (*F* (2, 12) = 104.3; *p* < 0.001) membrane polarization when compared to BOP 12 h and BOP 24 h groups.

### 3.3 Changes in ATP Production Following Bop in C6 and HTHU Cells

**Figure 3:**
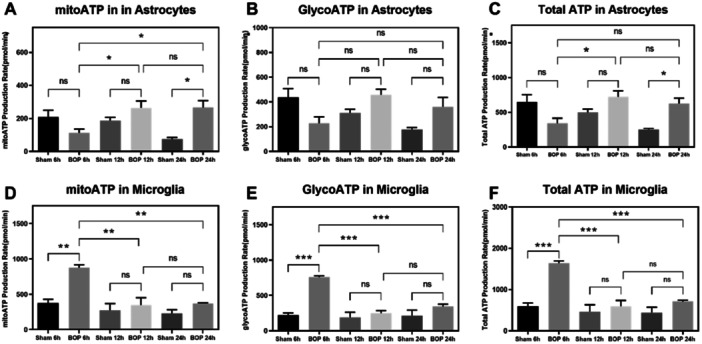
Effect of BOP on the cellular energetics of C6 and HTHU cells after blast overpressure in various time‐points (6, 12, and 24 h) using Seahorse XF 24 Analyzer. The histogram shows the mitoATP; GlycoATP and Total ATP in C6 and HTHU cells. Statistical difference between the cell groups were (*p < 0.05; **p < 0.005; ***p < 0.001) represented as Mean ± SEM (*n* = 3). (A) mitoATP in Astrocytes, (B) GlycoATP in Astrocytes, (C) Total ATP in Astrocytes, (D) mitoATP in Microglia, (E) GlycoATP in Microglia, and (F) Total ATP in Microglia.

The production of mitochondrial and non‐mitochondrial cellular ATP was measured using the Seahorse XFp Real‐Time ATP rate assay (Agilent Technologies, Santa Clara, USA). The results indicated a significant increase in the mitochondrial ATP (*F* (5, 12) = 5.992; *p < 0.05) levels in the BOP 24 h group of C6 cells when compared to sham‐exposed C6 cells (Figure 3). The non‐mitochondrial glycolytic and total ATP were significant in C6 cells compared to sham cells. Among BOP groups, the total ATP was significantly increased in 12 h post‐BOP (*F* (5, 12) = 6.340; *p < 0.05) when compared to the six h post‐BOP group. However, the BOP 6 h HTHU cells showed a significant increase in mitochondrial ATP (*F* (5, 12) = 12.02; *p* < 0.01), glycolytic ATP (*F* (5, 12) = 19.57; *p* < 0.001), and total ATP (*F* (5, 12) = 16.16; *p* < 0.001) when compared with the sham‐exposed cells. Compared to sham cells, these ATP levels were found to be nonsignificant in BOP 12 h and BOP 24 h group. Moreover, the total ATP (*F* (5, 12) = 16.16; *p* < 0.001), mitochondrial ATP (*F* (5, 12) = 12.02; *p* < 0.01), and the glycolytic ATP (*F* (5, 12) = 19.57; *p* < 0.001) were significantly reduced in BOP 12 h and BOP 24 h groups when compared to BOP 6 h group.

### 3.4 Western Blot Analysis Analysis of SIRT1 and SIRT3

**Figure 4:**
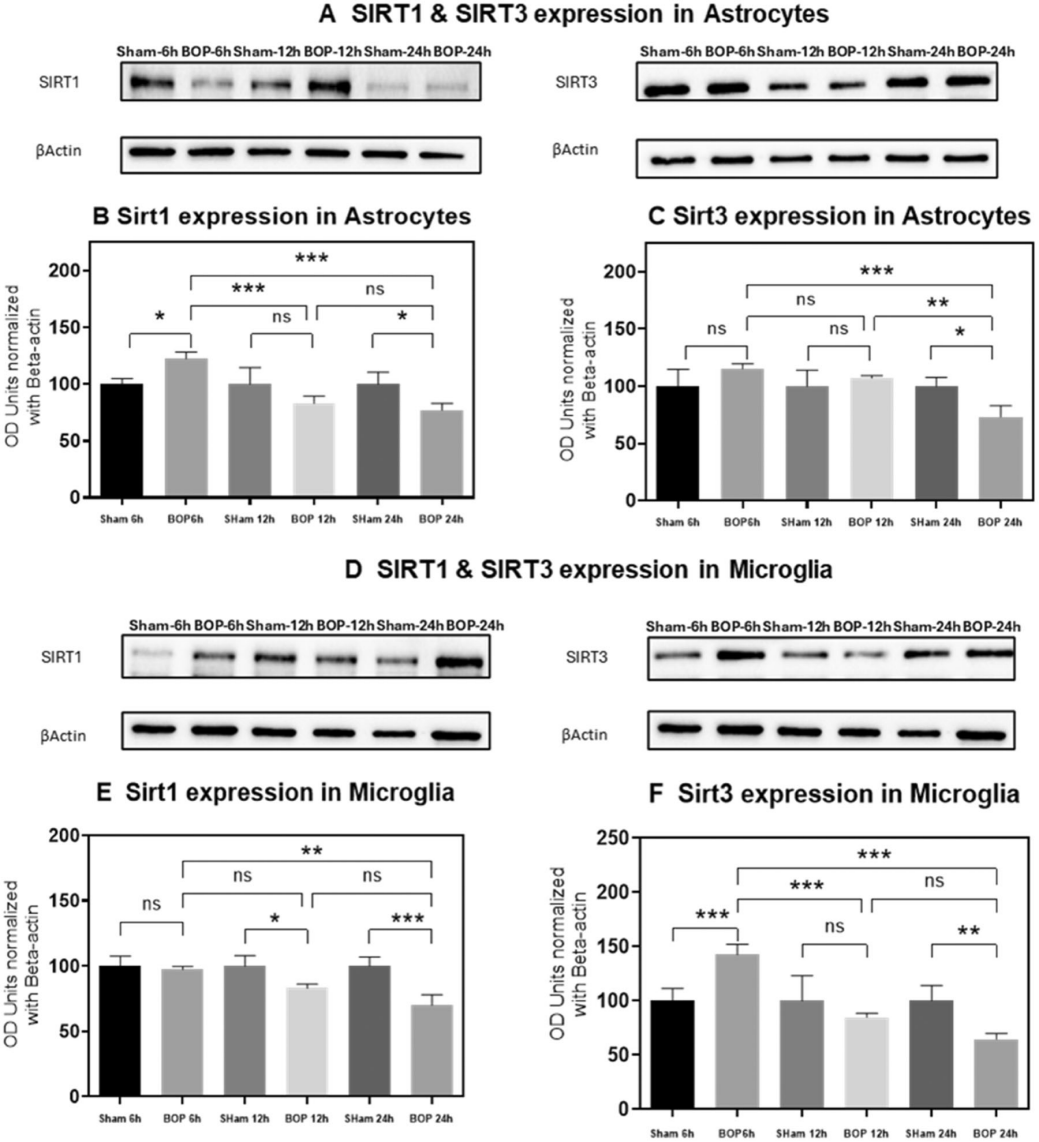
Assessment of SirT1 and SirT3 expressions by western blot analysis analysis in C6 and HTHU cells after BOP exposure. The immunoblots shows the protein expressions of SIRT1 and SIRT3 levels in (A) C6 and (D) HTHU cells normalized against the internal control β‐Actin. The histogram shows the semi‐quantitative protein expression of SIRT1 (B and E) and SIRT3 (C and F) levels in C6 and HTHU cells. Statistical difference between the cell groups were (*p < 0.05; **p < 0.005; ***p < 0.001) represented as Mean ± SEM (*n* = 3).

The western blot analysis demonstrated the changes in protein expression levels of SIRT1 and SIRT3 after BOP at various time points (Figure 4). The SIRT1 protein levels showed a significant change in BOP 6 h and BOP 24 h (*F* (5, 12) = 3.298; *p* < 0.05) groups when compared with sham‐exposed cells. Moreover, the Sirt1 protein level was reduced in BOP‐exposed cells in a time‐dependent manner. The Sirt1 level was significantly reduced in BOP 12 h and BOP 24 h (*F* (5, 12) = 3.298; *p* < 0.001) when compared to BOP 6 h group C6 cells. However, the SIRT3 expression was significantly increased only in BOP 24 h (*F* (5, 12) = 2.034; *p* < 0.05) cells when compared to sham C6 cells. Like SIRT1 expression, the Sirt3 protein levels also reduced time‐dependently. In C6 cells, the SIRT3 expression was found to be significantly reduced in BOP 24 h when compared to BOP 6 h (*F* (5, 12) = 2.034; *p* < 0.001) and BOP 12 h cells (*F* (5, 12) = 2.034; *p* < 0.005). Similarly, the Sirt1 and Sirt3 protein levels in the BOP‐exposed HTHU cells showed a time‐dependent reduction over various time points. The SIRT1 was found to be significantly reduced in BOP 12 h (*F* (5, 12) = 3.790; *p* < 0.05) and BOP 24 h (*F* (5, 12) = 3.790; *p* < 0.001) group HTHU cells when compared to sham group. Moreover, the Sirt1 level was significantly reduced in BOP 24 h (*F* (5, 12) = 3.790; *p* < 0.005) when compared to BOP 6 h HTHU cells. The SIRT3 level was found to be significantly increased in BOP 6 h (*F* (5, 12) = 4.013; *p* < 0.001) and the same was decreased considerably in BOP 24 h (*F* (5, 12) = 4.013; *p* < 0.005) HTHU cells when compared to sham exposed group. When comparing the BOP exposed groups, the BOP 12 h (*F* (5, 12) = 4.013; *p* < 0.001) and BOP 24 h (*F* (5, 12) = 4.013; *p* < 0.001) groups showed a significant reduction in the SIRT3 levels when compared to BOP 6 h HTHU cells.

### 3.5 BOP Exposure Results in Increased Par Expression

**Figure 5:**
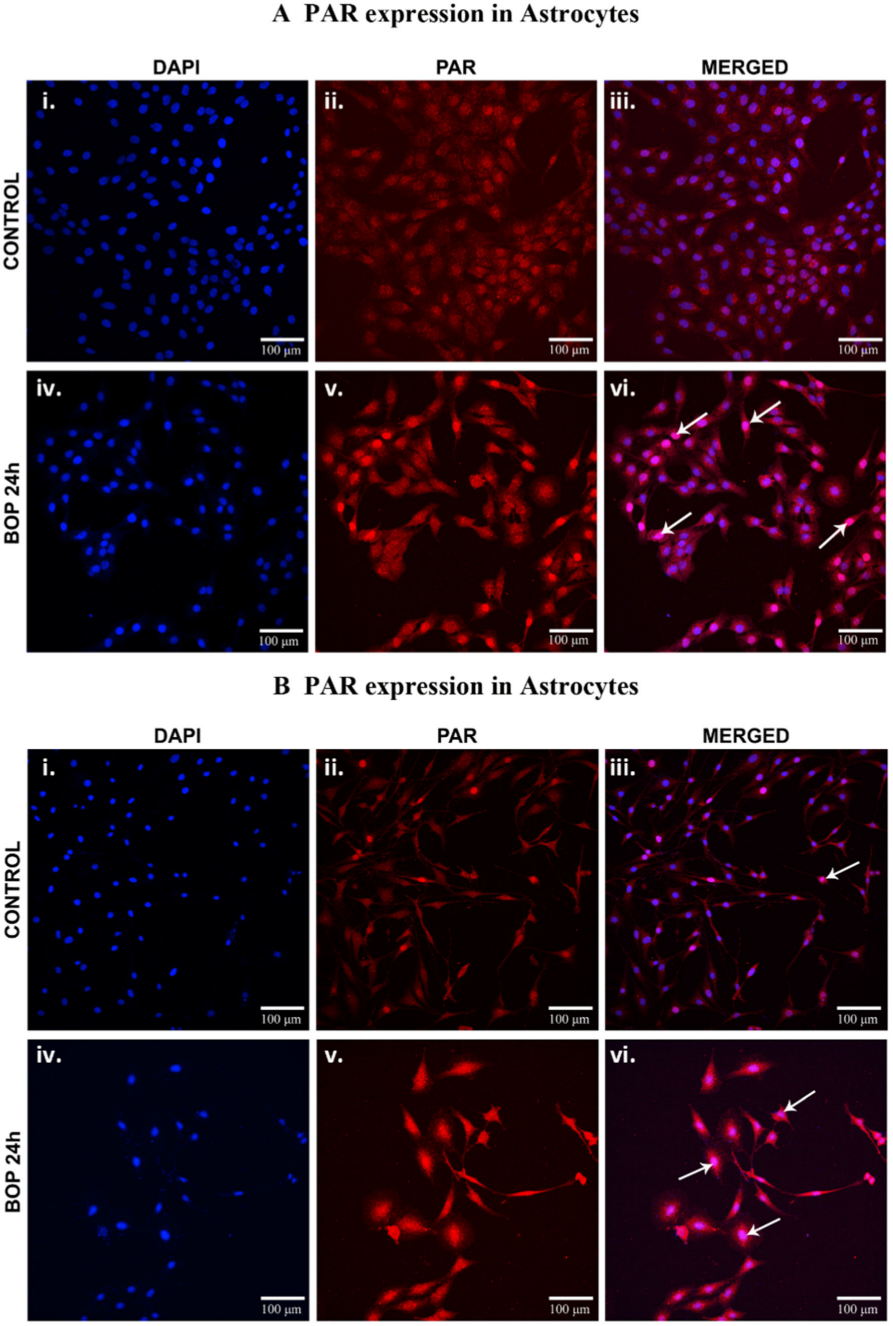
Qualitative immunofluorescence assessment of PAR expressions in C6 and HTHU cells after BOP exposure. The images display the cells expressing the nuclear stain DAPI (i and iv), PAR polymer (ii and v) and merged images (iii and vi) in control and 24 h post‐BOP (A) C6 and (B) HTHU cells. The white arrows indicate the cells expressing the PAR polymer with nuclear stain DAPI in C6 and HTHU cells.

The qualitative immunofluorescence staining procedure visualized the expression level of PAR. The results display the increased PAR expression in C6 and HTHU cells at post‐BOP 24 h time point compared to sham‐exposed cells (Figure 5).

### 3.6 BOP Modulates the mRNA Expressions of SIRT1, SIRT3, NRF2 and GCLC in C6 and HTHU Cells

**Figure 6:**
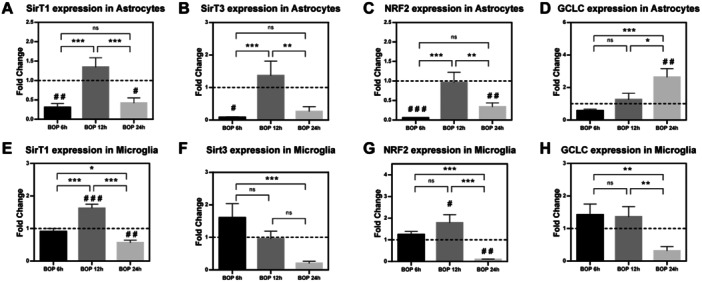
Reverse transcription‐quantitative polymerase chain reaction: SIRT1 (A and E), SIRT3 (B and F), NRF2 (C and G) and GCLC (D and H) mRNA expressions in (A) C6 cells (B) HTHU cells in various time‐points (6, 12, and 24 h). The values in the histogram represents the mean ± SEM (*n* = 3). Statistical difference between the various timepoint BOP groups (*p < 0.05; **p < 0.005; ***p < 0.001) and compared with sham group (#*p* < 0.05; ##*p* < 0.005; ###*p* < 0.001 vs. Sham) were represented as Mean ± SEM (*n* = 3).

The changes in mRNA expression levels of SIRT1, SIRT3, NRF2 and GCLC after BOP were analysed using the qPCR procedure in C6 and HTHU cells at various time points (Figure 6). The qPCR results have demonstrated a significant reduction in SIRT1 (*F* (5, 30) = 11.00; *p* < 0.005), SIRT3 (*F* (5, 30) = 6.644; *p* < 0.05), and NRF2 (*F* (5, 30) = 12.99; *p* < 0.001) levels in post‐BOP six h group when compared to sham in C6 cells. However, the expression was not significantly changed in the post‐BOP 12 h group when compared to sham‐exposed C6 cells. Moreover, the mRNA expressions of SIRT1 (*F* (5, 30) = 11.00; *p* < 0.05) and NRF2 (*F* (5, 30) = 12.99; *p* < 0.005) were significantly reduced with a significant increase in GCLC expression levels (*F* (5, 30) = 6.995; *p* < 0.005) in post‐BOP 24 h cells when compared to sham group. Among BOP‐exposed group C6 cells, the SIRT1 (*F* (5, 30) = 11.00; *p* < 0.001), SIRT3 (*F* (5, 30) = 6.644; *p* < 0.001) and NRF2 (*F* (5, 30) = 12.99; *p* < 0.001) levels were significantly increased in post‐BOP 12 h when compared to post‐BOP 6 h group. Surprisingly, the SIRT1 (*F* (5, 30) = 11.00; *p* < 0.001), SIRT3 (*F* (5, 30) = 6.644; *p* < 0.005) and NRF2 (*F* (5, 30) = 12.99; *p* < 0.005) expression were significantly reduced in post‐BOP 24 h group when compared to BOP 12 h C6 cells. Additionally, the GCLC expression was increased in a time‐dependent manner after BOP. The GCLC was found to be increased significantly in post‐BOP 24 h cells when compared to BOP 6 h (*F* (5, 30) = 6.995; *p* < 0.001) and BOP 12 h group (*F* (5, 30) = 6.995; *p* < 0.05) C6 cells. In HTHU cells, the mRNA expression levels of SIRT1 (*F* (5, 30) = 21.98; *p* < 0.001) and NRF2 (*F* (5, 30) = 11.10; *p* < 0.05) were found to be significantly increased in post‐BOP 12 h group. These SIRT1 (*F* (5, 30) = 21.98; *p* < 0.005) and NRF2 (*F* (5, 30) = 11.10; *p* < 0.005) expression were reduced significantly in post‐Bop 24 h group when compared sham exposed HTHU cells. Among the BOP‐exposed HTHU cells, the SIRT1 level was significantly increased in the post‐BOP 12 h timepoint (*F* (5, 30) = 21.98; *p* < 0.001) when compared to the BOP 6 h group. But the expression levels of SIRT1 (*F* (5, 30) = 21.98; *p* < 0.001) and NRF2 (*F* (5, 30) = 11.10; *p* < 0.001) was reduced significantly in BOP 24 h group when compared to BOP 12 h cells. However, the SIRT3 expression was significantly reduced in BOP 24 h HTHU cells (*F* (5, 30) = 4.821; *p* < 0.001) compared to the BOP 6 h group. Additionally, the NRF2 and GCLC expression levels were found to be significantly decreased in post‐BOP 24 h timepoint when compared to BOP 6 h and BOP 12 h group HTHU cells.

## 4 Discussion

The cell death mechanisms of TBI, especially the impact injury (blunt), were investigated widely [31, 32]. However, the cellular mechanism of neurodegeneration post‐blast (nonimpact) injury remains elusive [33]. demonstrated the blast‐induced activation of microglia and astrocyte organotypic hippocampal cultures [33]. Bricker‐Antony and Rex 2014 showed that cell death after blast trauma in the eyes was primarily non‐apoptotic [34]. In our recent study on in vitro models (astrocytes and microglia) of low‐frequency blast overpressure, PARP overactivation was evidenced by the perturbation of mitochondrial energetics in microglia and astrocytes. We found that low‐frequency BOP‐induced trauma results in over‐activation of PARP, resulting in trans‐PARylation with increased expression of mitochondrial electron transport complexes and reduced basal mitochondrial respiration, resulting in increased apoptosis with marginal necrosis. In another report on impact trauma, we found that inhibition of PARP using a known potent inhibitor, 3‐aminobenzamide (3AB), attenuated the trauma‐induced oxidative stress by increasing the master redox regulator glutathione through increasing the expression of the rate‐limiting enzyme glutamate‐cysteine ligase catalytic (GCLC) unit in the process of glutathione synthesis [35]. NAD^+ (^nicotinamide adenine dinucleotide) is a common substrate for both PARP and Sirtuins, with NRF2 being one of the common deacetylation targets of Sirtuins. Here, we investigated blast injury's effect and modulation on the PARP‐SIRT‐NRF2 axis. Zander et al. 2015 studied the impact of blast injury on neuronal populations [13]. However, non‐neuronal glial cells play a critical role in mitochondrial dysfunction, neuroinflammation, oxidative stress, and blood‐brain barrier disintegrity post‐TBI [36]. Hence, to further probe the cellular mechanism of blast injury, here we investigated the impact of the blast overpressure on the modulation of the antioxidant defense system in in‐vitro cultures of microglia (HTHU) and astrocytes (C6).

Post‐BOP, we found that reactive oxygen species increased significantly in both astrocytes and microglia, which is due to the loss of mitochondrial membrane potential (∆Ψ_m_) as evident from the increased ratio of JC10 aggregates to JC10 monomers [37]. This loss or reduced ∆Ψ_m_ is attributed to the perturbated mitochondrial permeability transition leading to the release of cytochrome c, AIF, and other factors of cell death induction [38]. As MPT lays the ground for ATP synthesis, we determined the ATP generation of both cells post‐BOP from both mitochondria and glycolysis. In astrocytes, post‐BOP, though, the mitochondrial and glycolytic ATP levels reduced abruptly; the ATP levels were recuperated at 24 h. Similar to this differential response of astrocytes in recuperating the mitochondrial ATP, Almeida et al. 2001 reported the differential response of astrocytes in bioenergetic recuperation through glycolytic ATP. However, under BOP insults, we found that the recovery was observed in both sources of mitochondria and glycolysis. It has been postulated that ATP release from injured tissue establishes the long‐range extracellular ATP gradient for the chemotaxis of the remote microglia to reach the wounded site to clear debris (Dou et al. 2012) in addition to ATP being a mediator of calcium signaling between astrocytes and microglia [39]. On the contrary, in microglia, the ATP levels were found to be increased in the acute phase of post‐BOP, but the ATP levels were reduced or found to be the same as controls. Evidence indicates that microglia use glycolysis for energy production at pro‐inflammatory conditions, and at anti‐inflammatory conditions, microglia prefer oxidative phosphorylation (OXPHOS) for energy production [40]. At present, though, ATP levels from both sources were elevated post‐BOP, an increase of ATP by glycolysis more significant than the OXPHOS source, indicating the proinflammation post‐BOP.

**Figure 7:**
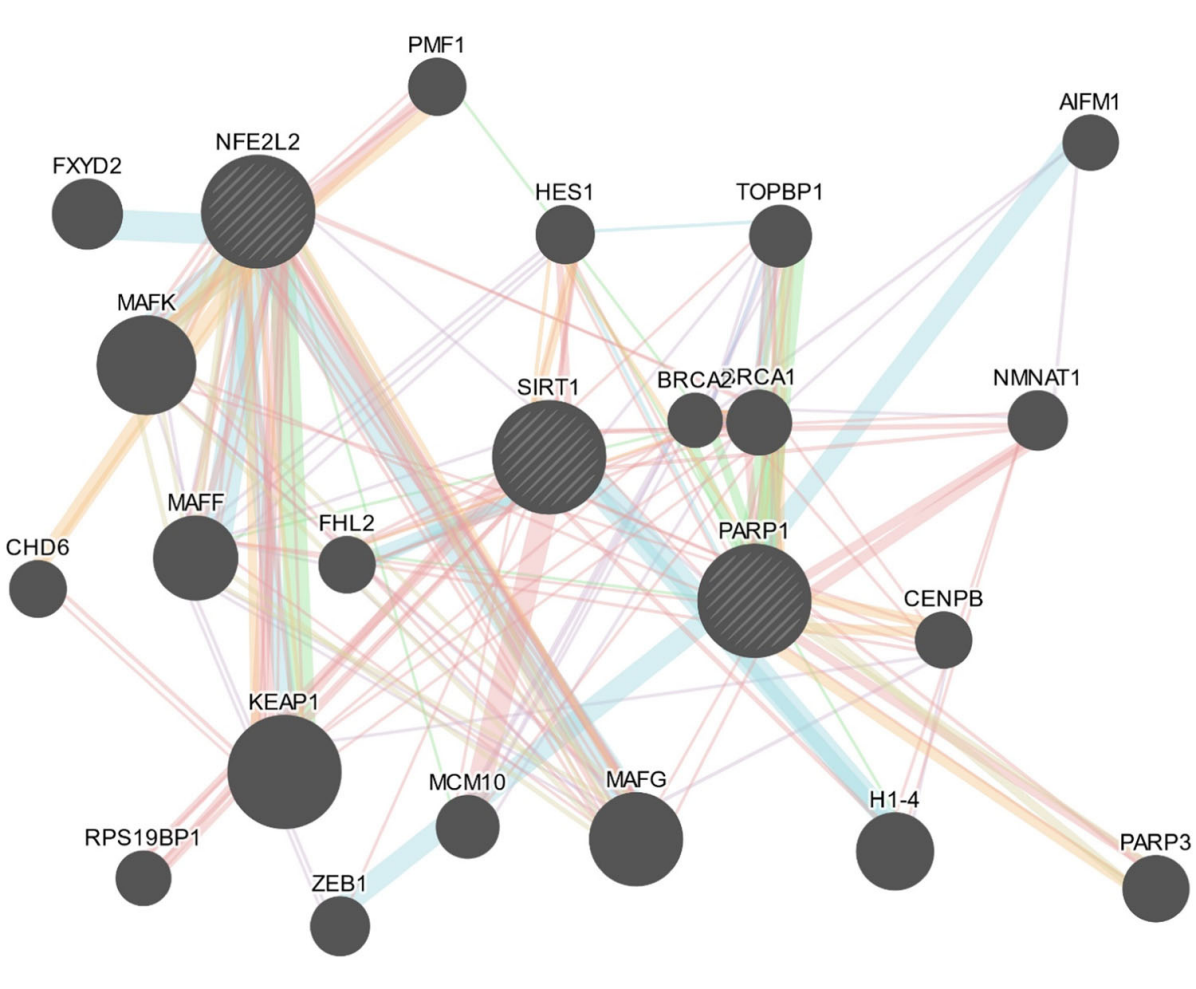
Potential interaction between PARP1, NFE2L2 and Sirt1 as determined from Genemania.

Like our earlier report, we confirmed the BOP‐induced PARP over‐activation through increased formation of PAR polymers on several proteins in astrocytes and microglia post‐BOP. The trans‐PARylation process utilizes NAD^+^ [41]. Reports indicate that under the conditions of glucose withdrawal, cells rely on mitochondria for ATP through activation of SIRT1 and SIRT3 [42, 43]. However, NAD^+^ being a substrate for sirtuins to deacetylate several targets of transcriptional regulators [44], PARP over‐activation directly affects the Sirtuins activity, which was reflected from the reduced gene and protein expression of SIRT1 and SIRT3 post‐BOP. This finding is corroborated with (Figure 7) earlier evidence of the inhibition of the capability of SIRT1 to deacetylate its targets as a result of rapid depletion of NAD+ levels by PARP overactivation [45, 46] and increased SIRT1 activation through PARP1 inhibition [47]. As Sirtuins regulate the transcriptional activity of NRF2 (Kratz et al. 2021), the downregulation of SIRT1 and SIRT3 is reflected in the gene expression of NRF2 as evidenced by the decreased levels post‐BOP. Similar findings were also observed in‐vivo study [48]. As NRF2 binds to the cis‐acting element of ARE, this downregulated SIRT/NRF2 pathway seems to suppress the NRF2/ARE (Antioxidant response element genes) [49] which was evidenced by the downregulated expression of the rate‐limiting enzyme of glutathione synthesis, glutathione cysteine ligase catalytic unit (GCLC) enzyme in microglia. NRF2 is a key regulator of Glutathione synthesis through glutathione cysteine ligase enzyme in which GCLC is part of the heterodimer along with GCLM (modifier unit) [50, 51]. These results were corroborated with another study in which GCLC was downregulated by siRNA specific for Sirtuins [52]. However, the GCLC gene expressions were found to be upregulated in astrocytes which agreed with increased Total ATP levels. It is unclear how GCLC levels and ATP levels were increased post‐BOP in astrocytes in contrary to microglia. However, the differential expression of GCLC in microglia and astrocytes needs to be confirmed with GCL activity (K_cat_) [53].

Overall, this differential response of astrocytes and microglia in response to BOP warrants further investigation in terms of other effectors and under different parameters of trauma specifications such as blast repetition, intensity, inter‐blast intervals. Thus, the study of the effect of BOP on the modulation of the PARP‐SIRT‐NRF2 axis provides mechanistic insights on blast‐induced glial pathomechanism.

## Author Contributions

Vijaya Prakash Krishnan Muthaiah has conceptualized and administered the project in addition to resource support and manuscript preparation. Kathiravan Kaliyappan has performed most of the in vitro experiments such as cell viability, flow cytometry, western blot analysis, mitochondrial respiration, and quantitative PCR. Krishnamoorthy Gunasekaran has provided technical consultation and helped with the data analysis. Supriya Mahajan has helped in the data curation, manuscript preparation, and critical reading of the manuscript.

## Ethics Statement

This study utilizes in vitro cell lines as an experimental platform to investigate the signaling mechanism following blast injury. Hence, this study does not warrant approval from Institutional Animal Care and Use Committee and Institutional Review Board.

## Conflicts of Interest

The authors declare no conflicts of interest.

## Data Availability

The data that support the findings of this study are available from the corresponding author upon reasonable request.
